# Prosthetic rehabilitation of a patient with cleidocranial dysplasia using dental implants—a clinical report

**DOI:** 10.1186/s40729-020-00287-7

**Published:** 2021-01-22

**Authors:** Sigmar Schnutenhaus, Werner Götz, Ralph G. Luthardt

**Affiliations:** 1 Department of Prosthetic Dentistry, Center of Dentistry, Ulm University, Ulm, Germany; 2 Center for Dentistry Dr. Schnutenhaus MVZ GmbH, Breiter Wasmen 10, 78247 Hilzingen, Germany; 3 Department of Orthodontics, Oral Biology Laboratory, University of Bonn, Bonn, Germany

**Keywords:** Cleidocranial dysplasia, Rare diseases, Osseointegration, Implant restoration, Immediate loading

## Abstract

Adult patients with oral manifestations of untreated syndromic malformations usually exhibit a high degree of suffering. In this clinical report, we describe the implant-supported prosthetic treatment of a patient with cleidocranial dysplasia, a rare autosomal-dominant inherited malformation syndrome. Therapy for oral manifestations of cleidocranial dysplasia should be started in early childhood; however, the 26-year-old patient in the present study had not undergone orthodontic therapy in childhood. The treatment measures performed prior to this study were limited to the removal of several permanent teeth. Surgical pretreatment, placement of six implants each in the maxilla and mandible, and prosthetic restoration are described. The implantation was guided using a three-dimensional template. Long-term immediate temporary restoration and immediate loading of the implants were performed. The definitive prosthetic restoration was completed using fixed, acrylic resin-veneered screw-retained fixed dental prostheses. The clinical and radiological parameters observed in this case suggest that surgical and prosthetic procedure concepts from implantology can be adopted for patients with CCD.

## Background

Cleidocranial dysplasia (CCD) is a rare autosomal dominant malformation syndrome, with a prevalence rate of 1:1,000,000 and no correlation with gender or ethnic origin [1]. CCD was first described by Pierre Marie and Paul Sainton in 1897 [2]. Various mutations in CBFA1/RUNX2, a transcription factor on chromosome 6p21, have been identified as the cause [3]. This transcription factor is responsible for the differentiation of precursor cells into osteoblasts and regulates the differentiation of chondrocytes in the growth plate [4]. Hypoplastic or absent clavicles and changes in the skull are the most prominent signs of CCD. In addition, brachycephaly, a depressed nasal bridge, and open or delayed closure of the fontanelles also occurs [5]. Other disorders can occur in the chest, spine, pelvis, and extremities [6]. The general health of patients with CCD is usually good, and there are no intellectual limitations [7]. Dental abnormalities are one of the main manifestations of CCD [8]. CCD patients often have an excess number of teeth (hyperdontia), delayed eruption of teeth in the primary and permanent dentition, or retention of teeth [9]. This is accompanied by a misalignment of the teeth and consequent malocclusion. However, phenotypic analysis has also shown that the severity of malformations in the skeletal system may not necessarily correlate with that of the disruption of tooth development [10]. A hypoplastic maxilla and associated pseudoprognathia can also be a skeletal sign of CCD [11]. In these cases, the maxilla may be characterized by reduced anterior and posterior height, and the palate may be high, narrow, and strongly arched [12]. In the mandible, parallel rami, narrow, distally rounded coronoid processes, and changes to the condyles have been described [13]. In addition, radiological assessments of the alveolar bone show a dense and compact structure [14]. Due to the predominantly irregular position and shape of the teeth, chewing disorders and an oral appearance that may cause psychological stress are the patients’ chief complaints [15]. In patients with CCD, the focus of treatment is on achieving optimal masticatory function and correcting the esthetic appearance in young adults [16]. As the care of such patients is complex, a multidisciplinary treatment strategy is typically used for these patients. In such cases, psychological care is also worth considering, as some patients with CCD must cope with severe disfigurement in a world focused on physical appearances. In addition, changes brought about by dental measures that alter patients’ self-image may not be easy to manage.

The correction of malocclusion is achieved through a combination of surgical, orthodontic, and possibly prosthetic treatment measures [15, 17]. Surplus and retained teeth must often be extracted [18]. Surgical exposure of impacted teeth for subsequent orthodontic adjustment or surgical adjustments and autografting has also been described [19]. Permanent teeth with an abnormal shape can be used as abutments for prostheses [20] or must be removed [21]. Various concepts have been described with the objective of achieving normal masticatory function and esthetics [22]. The consensus of these treatment strategies is that early diagnosis is necessary, and treatment should be started early in childhood and adolescence [23]. If orthodontic therapy is not successfully completed or if the patient does not undergo dental treatment until adulthood, a prosthesis-based treatment plan must be implemented. For this purpose, teeth that show a favorable prognosis and are in a position suitable for prosthetic purposes can be used as abutments for fixed or removable dentures [24]. Alternatively, complete prostheses can be considered for a socially acceptable solution. In addition, removable dentures can often be used to help restore the lower-face profile and lip support [25]. Temporary removable dentures are also useful in patients who are unable to receive implants due to their age and incomplete jaw growth [26]. On the other hand, various case reports have documented the success of dental implants for anchoring removable or fixed dentures in CCD patients [24, 27].

The patient presented here showed all the clinical symptoms of CCD. The aim of this case presentation is to illustrate the treatment procedure for a late restoration, using implant-retained fixed dentures. Different treatment alternatives are discussed, and recommendations for decision-making are provided.

## Clinical report

The male patient in this study was 26 years old at the first presentation and showed definite symptoms of CCD. The patient was aware of his diagnosis, as he had been diagnosed by a pediatrician during childhood. Moreover, family history indicated that his father also had CCD. The primary reason for his visit was severe pain originating in the carious upper right first molar. After the initial pain treatment, the possibilities of creating a normal masticatory function and a natural-looking esthetic appearance were discussed. The unesthetic positioning of the patient’s teeth caused him tremendous amount of psychological strain, and consequently he avoided smiling. The patient’s general medical history showed no abnormalities, and there were no diseases that would contraindicate comprehensive dental rehabilitation. An initial alternative therapy, such as orthodontic treatment, had not been performed.

**Fig. 1:**
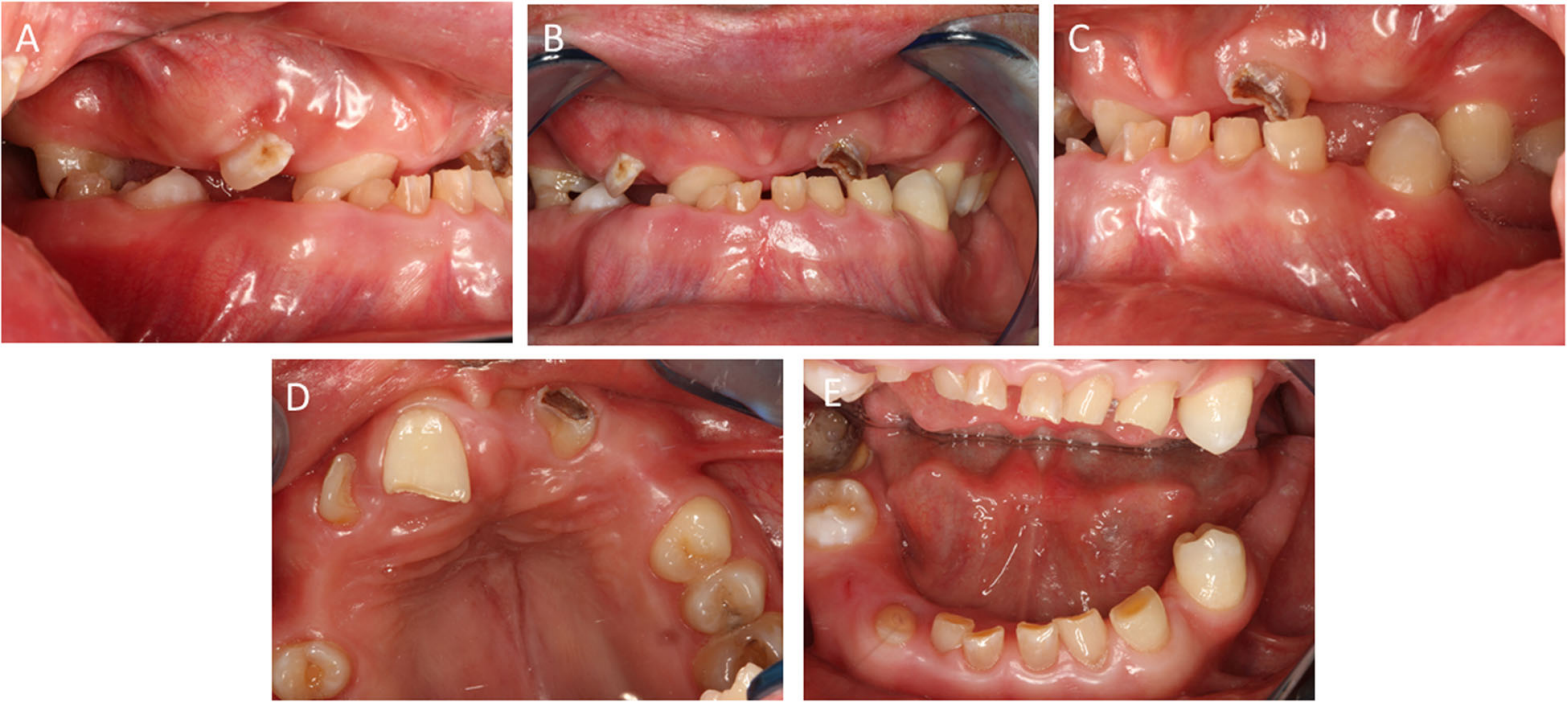
**a**–**e** The initial findings in a 26-year-old patient with CCD. CCD, cleidocranial dysplasia

**Fig. 2:**
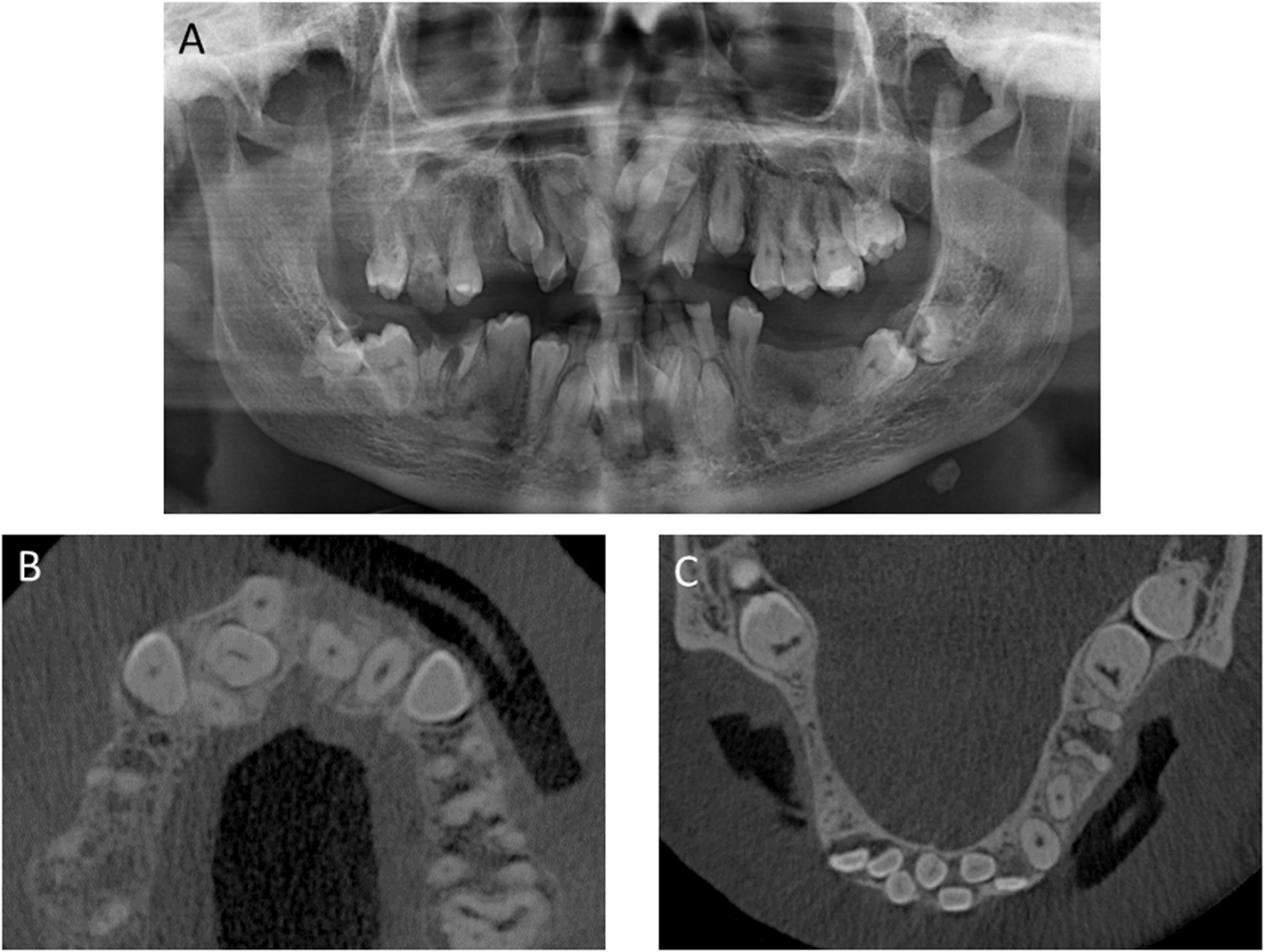
**a** The OPG and **b**, **c** excerpts from the CBCT scan. Typical CCD findings can be seen: retained teeth, jaw malposition, and bony variations. OPG, orthopantomogram; CBCT, cone-beam computed tomography; CCD, cleidocranial dysplasia

**Fig. 3:**
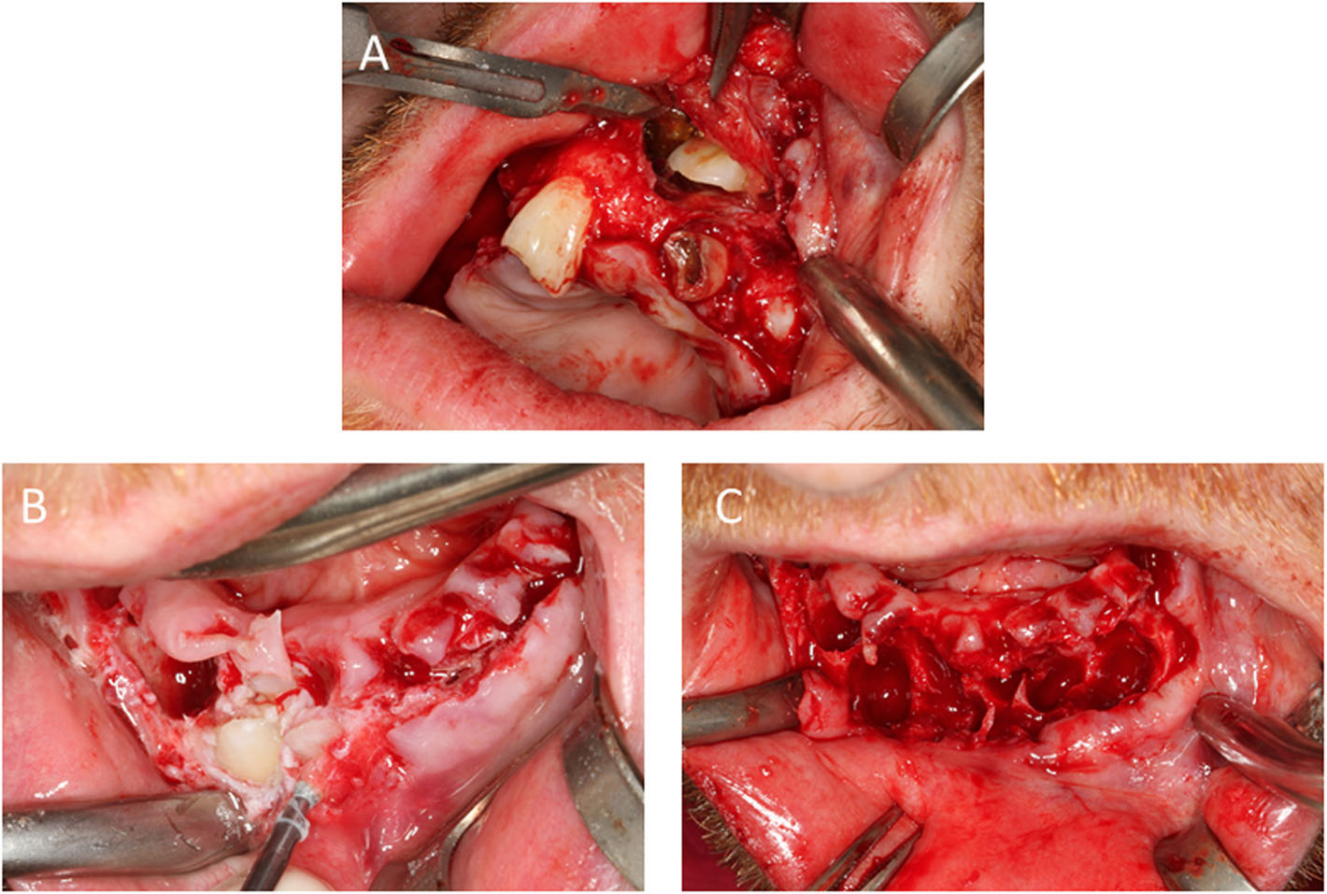
**a** Removal of tooth 13 with the retention cyst. **b** Exposure of the impacted teeth. **c** Defect in the mandible after tooth removal

After controlling the pain and thorough cleaning of the teeth, a definitive assessment was performed (Fig. 1). Teeth 1, 5, 16, 19, 20, and 21 were missing. Teeth 6, 8B, 9A, 9B, 11, 15, 18, 19, 22–28, 31, and 32 were retained and displaced. Teeth 3, 7, and 19 were decayed. Therefore, the right maxillary molar [3] also had to be removed prior to any additional treatment. The primary teeth present were C and M–Q. Other findings included enamel hypoplasia, abnormal size and structure of the erupted teeth, a high palate with a deep furrow in the midline and a protrusive biting position. The periodontal examination did not reveal any abnormalities. There was significant dysfunction due to an open bite; only antagonist pair 2/31 showed static and dynamic occlusal contacts. The radiographs on admission (Fig. 2a–c) showed the typical signs of CCD—parallel wall alignment of the two sides of the mandible and narrow, pointed coronoid processes. In addition, the maxillary sinus on each side was hardly pneumatized, and a cyst lesion with a diameter of 18 mm was detected radiographically between teeth 8 and 10. The numerous non-erupted teeth (Fig. 3) and cysts in relation to the maxillary and mandibular anterior teeth made extensive surgical pretreatment necessary.

The treatment alternatives were explained to the patient in detail. Three possibilities were presented to him: (a) removal of all teeth and insertion of removable complete dentures; (b) preservation of a few strategic abutment teeth and insertion of dentures with double crowns as anchoring elements. In this plan, implants for strategic pillar multiplication were also suggested. (c) An implant-supported prosthetic restoration, either fixed or removable, after extraction of all teeth. The patient wanted a fixed, implant-supported restoration. This decision was influenced by his father’s treatment 5 years earlier, in which an implant-supported restoration was inserted. From a prosthetic and periodontal viewpoint, preservation of teeth 4, 12, 13, and 14 would have been possible. If the bite had to be changed, a crown restoration would have been necessary. The patient refused to receive any teeth because they “do not belong to him”.

The patient requested the removal of all his teeth and a long-term provisional restoration with complete denture prostheses. The surgical procedure was performed 1 year after the initial presentation. The extraction of the teeth, removal of the cysts, and osteotomy of the maxilla and mandible were performed under local anesthesia. The bone defects were completely covered after thorough curettage and complete removal of the cystic tissue. Teeth 17 and 18 were retained at this time, in accordance with the patient’s request. After removal of the required teeth, the pronounced defect situation became apparent. However, the defects were not filled with autologous bone or bone-substitute materials. Removable complete dentures were inserted as temporary restorations. These temporary prostheses were also necessary to study the patient’s occlusion. Since there was no indication of the position of the occlusal plane, the lengths and widths of the prosthetic teeth were taken from the cone-beam computed topography (CBCT) by measuring the sizes of the permanent teeth.

The function was checked and corrected several times during the healing period.

The new occlusion established in this way served as a template for the fabrication of the drilling template and for transferring the jaw relation during the fabrication of implant-supported long-term temporary restorations.

During the healing period of 6 months, the alveolar ridges atrophied considerably. Likewise, CCD-typical micrognathia and pseudoprognathia were seen in the edentulous state. A new CBCT scan was performed for design purposes. Casts of the jaws were mounted on an articulator in relation to the cranial base. Digital teeth set-ups were superimposed with the CBCT in an implant planning software (coDiagnostiX, Dental Wings, Inc., Montreal, Canada). A prosthetically oriented three-dimensional design of the implant positions was created, and a drilling template was constructed.

We planned to insert six implants each in the maxilla and mandible. The implant positions were determined by template-guided pilot drilling. A transgingival pilot hole was drilled and used to determine the exit point and axial direction of the implant. The prosthetic plan involved using screw-retained fixed dental prostheses (FDPs) in the maxilla and mandible. A complete preparation of the implant beds using the template was not planned because of the restricted mouth opening. After the pilot drilling, the jawbone was exposed, and complete freehand preparation was performed. Because of the low radiological density of the bone structure, implants with an expected high primary stability (BLX implant, Straumann AG, Basel, Switzerland) were inserted. All implants had a diameter of 4.5 mm. The lengths were 8–14 mm, depending on the available bone.

**Fig. 4:**
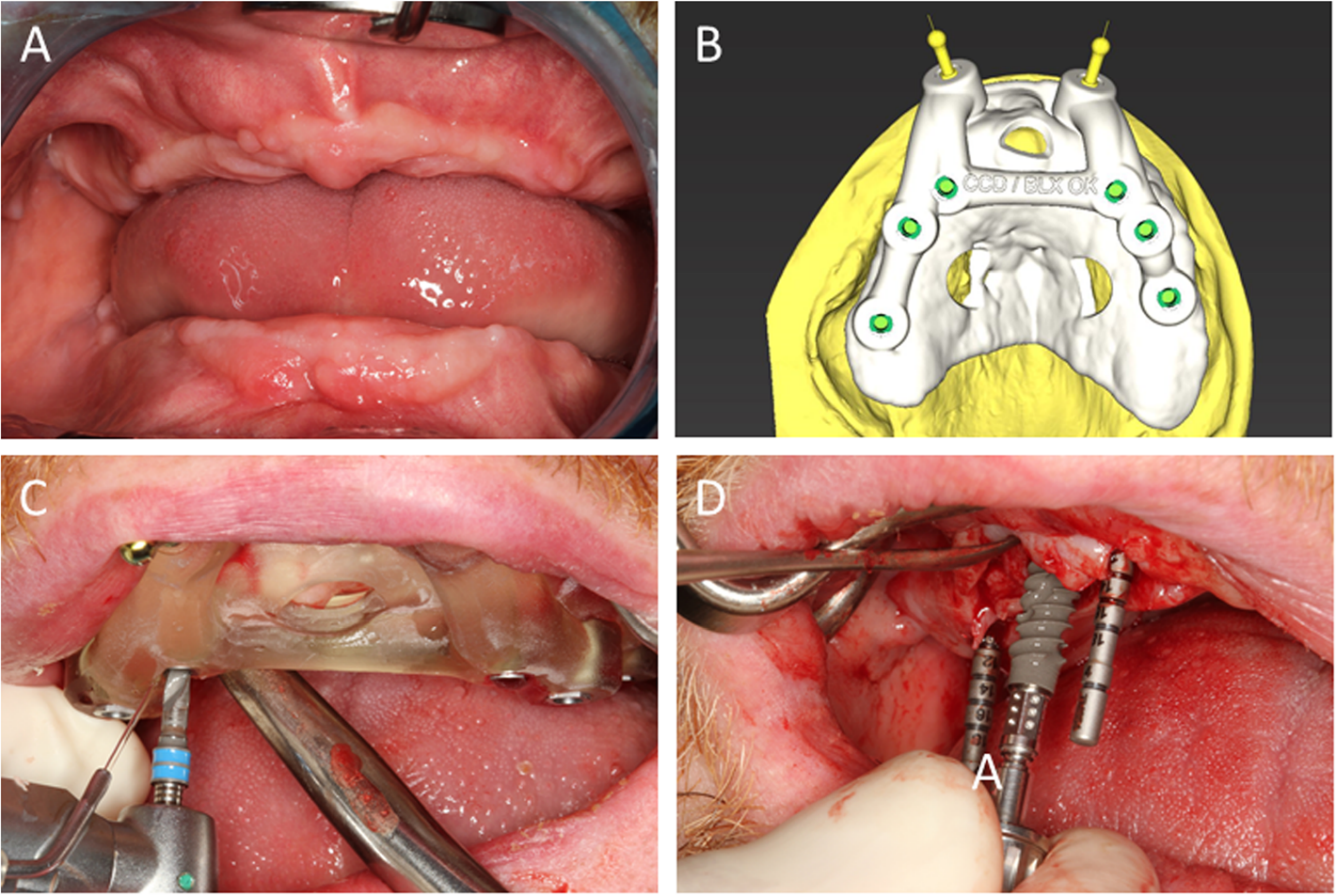
**a** The condition 6 months after surgical removal of all teeth. **b** Construction of the drilling template. **c** Stabilization of the template using transverse pins. Pilot holes being drilled using the template. **d** Further preparation and implant insertion being performed in a conventional manner

All implants could be inserted in a primarily stable manner (insertion torque > 35 Ncm). Because of the adequate bone quantity and computer-supported implant position planning, no augmentation measures had to be implemented (Fig. 4a–d). Thus, the planned temporary restoration could be performed immediately after implant placement. For this purpose, abutments for screw-retained FDPs were attached to the implants. Owing to the design and template-guided insertion, only straight abutments could be fitted. Impressions of the implants were taken after suturing. After the facebow transfer and bite registration, the implants remained unsupported for 10 h. During this time, long-term provisional fixed dental prostheses were fabricated using computer-aided design/computer-aided manufacturing (CAD/CAM).

The plaster models were digitized (3Shape Scanner D 700 3Shape A/S, Copenhagen, Denmark). The occlusion was adjusted with a postoperative silicone bite. In addition, it was checked and corrected using the previous full dentures. Since implantation was carried out in a minimally invasive manner, these could be repositioned relatively precisely on the plaster models. The long-term temporary implant-supported dentures were then designed using the corresponding CAD software. The FDPs were milled from multicolor polymethylene methacrylate (PMMA) plastic. The basal part was then covered with a gum-colored plastic. After bonding the titanium adhesive bases, the static and dynamic occlusion of the dentures was corrected on the articulator. This procedure requires close coordination between the dental team and the dental laboratory. The procedure and processes must be coordinated very well in order to ensure fast production.

**Fig. 5:**
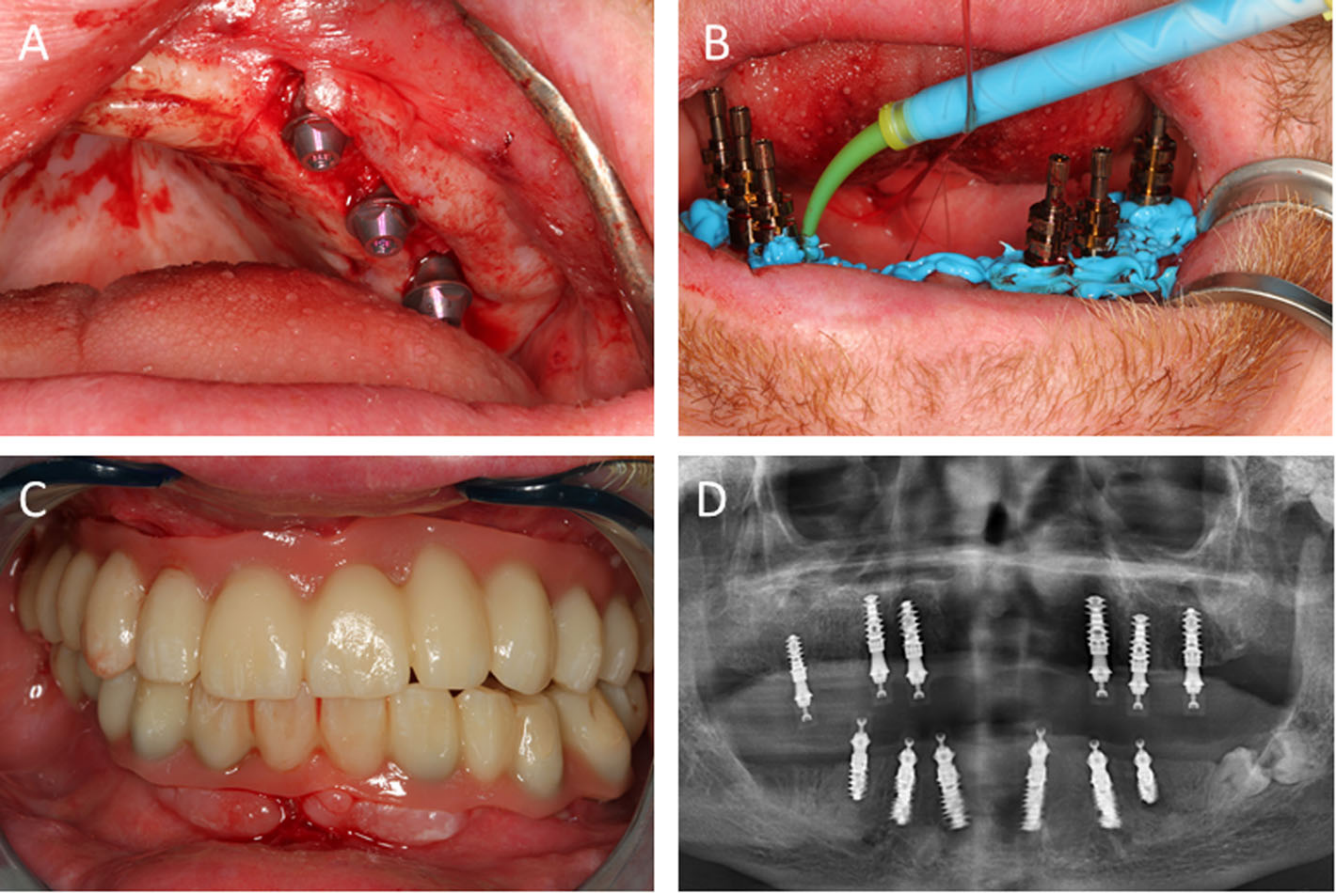
**a** The implants are restored with abutments for screw-retained FDPs. **b** An impression of the area being taken after suturing. **c** The fixed long-term provisional dental prostheses placed with screws 10 h after implantation. **d** A radiographical control image obtained after implantation

The two FDPs were inserted the following day (Fig. 5a–d). The patient was kept on soft food for 6 weeks. An analgesic (ibuprofen 500 mg) was prescribed, and the patient was instructed to take it at his own discretion within the prescribed maximum dose.

After the implants healed for 4 months, the final prosthetic restoration was performed. The healing period passed without any special incidents. However, functional adaptation of the denture was observed during this phase. There was no discomfort related to either the implants or the prosthetic restoration. After removing the temporary restoration, we checked whether the final abutments were tightened to a torque of 35 Ncm. An open-tray impression of the implants was taken. Bite registration was performed using the long-term temporaries after fabricating the cast. This allowed the tested position to be safely transferred to a fully adjustable articulator.

**Fig. 6:**
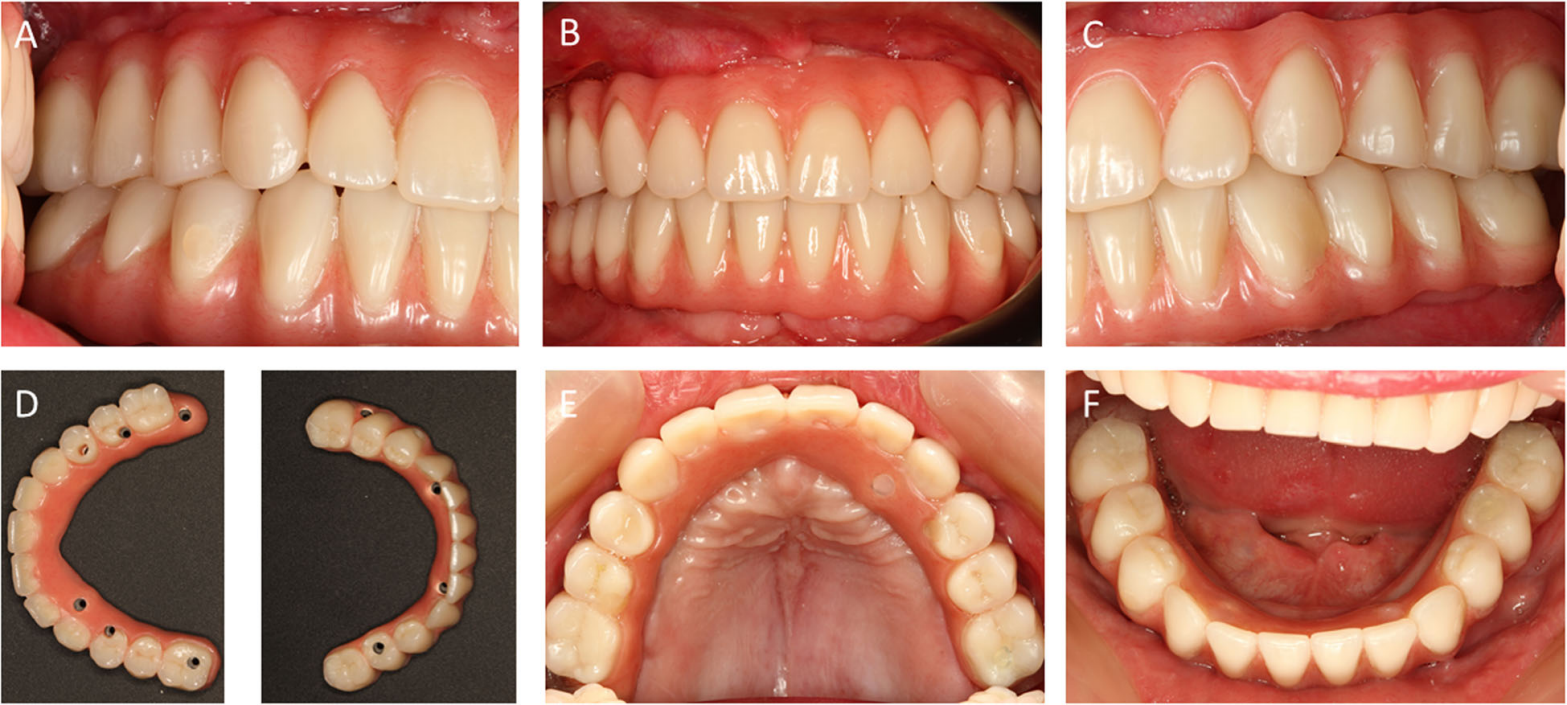
**a**–**e** Images of the dental prostheses after completion and integration

**Fig. 7:**
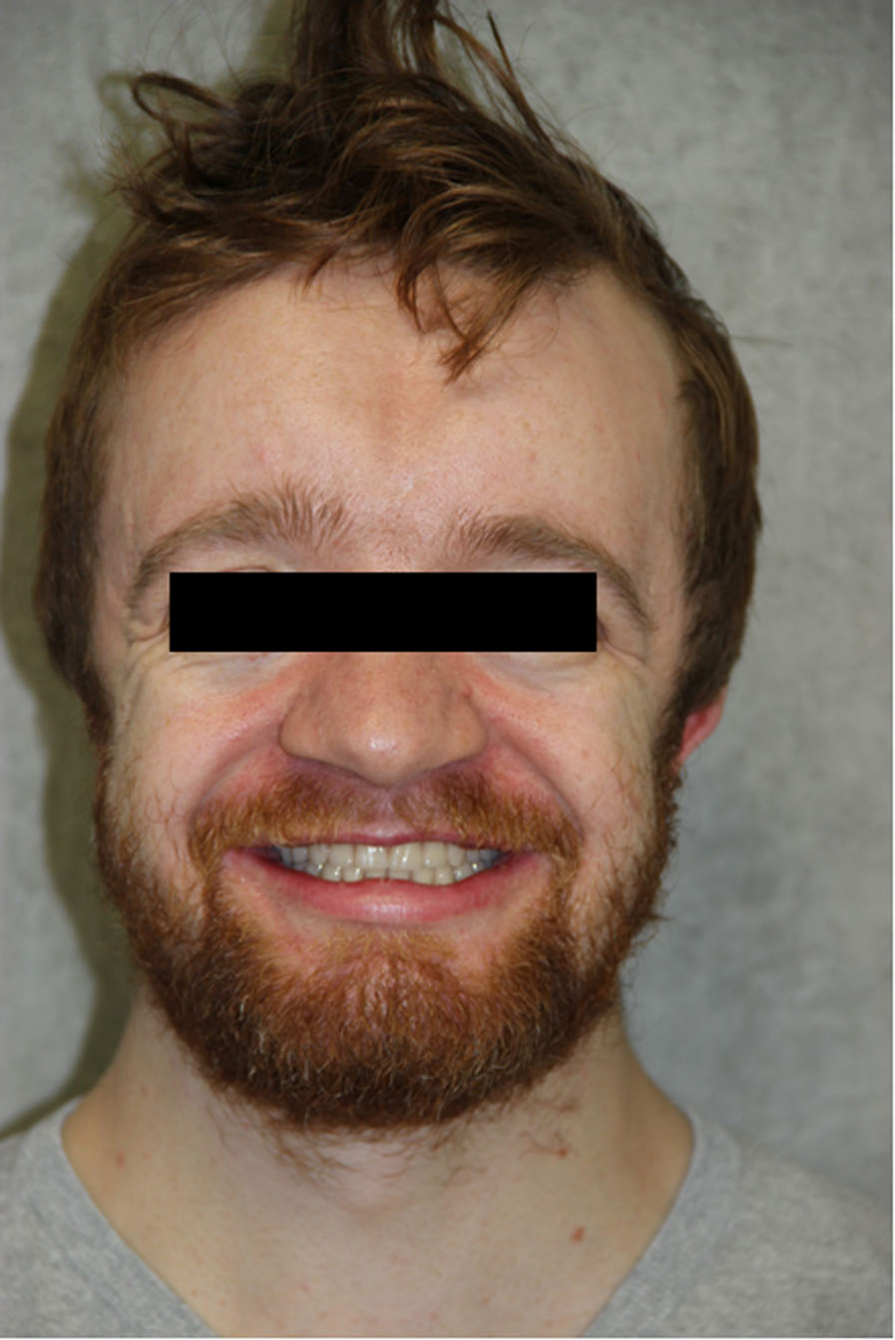
Final frontal image after insertion of the fixed dental prostheses

Trial fitting of the framework was performed using a wax setup positioning of the teeth. As no concerns arose regarding static and dynamic occlusions, esthetics, or phonetics during this check, the anatomically contoured metal alloy frameworks were veneered using acrylic resin (PhysioStar NFC, Candulor, Roelasingen-Worblingen, Germany). The FDPs were fitted in the following session (Fig. 6a–f). The screw channels were closed with Teflon tape and a light-curing composite material. By raising the vertical dimension between the opposing arches, a satisfactory profile was created. In addition, a prosthesis-based adjustment for the micrognathia of the maxilla and pseudoprognathia was achieved with the denture. At the conclusion of the procedure, the patient reported feeling very satisfied with the functional and esthetic outcome of his denture (Fig. 7). To date, the patient has been followed for 9 months, and no complications have been reported.

## Discussion

The treatment of patients with CCD should begin in early childhood and adolescence; this can only be achieved by early diagnosis. Although various treatment strategies for CCD have been described in the literature, the consensus is that therapy should be started at an early age. Early orthodontic therapy can help reduce malocclusion. With orthodontic adjustment of teeth, particularly in height and direction, teeth can be preserved, and the foundation for a later prosthetic restoration can be created [15]. If treatment cannot begin during childhood, prosthetic rehabilitation is a successful treatment method and can be performed with complete dental prostheses [28]. Therapy in adulthood must also determine whether patients can expect a result that provides therapeutic relief with the integration of permanent teeth. In the present case, orthodontic therapy was ruled out, due to the small number of teeth that was sustainable, the age of the patient, and the clear rejection by the patient.

Creating sufficient function and an improved esthetic situation can be achieved even with simple means and limited financial resources. In addition, restoration with the integration of implants is possible. The advantages of implant therapy compared to conventional total prosthetic restoration include prevention of bone resorption, the possibility of integrating fixed or partly removable dentures, and stabilizing a removable denture. The decision of whether to use a fixed or removable implant-retained denture must be made by the patient and surgeon in advance. In making this decision, advantages and disadvantages, such as chewing comfort, speech formation, cleaning possibilities, psychological acceptance, and esthetics, should be discussed [29]. Further, the cost differences in the types of restorations resulting from the number of implants and complexity of the dental technical services should be discussed with the patient [30]. When deciding on an implant-supported denture, the prognosis should also be assessed. Tooth preservation must be weighed. The preservation of individual teeth can also have a positive impact on the tactility and neuromuscular function of the chewing organ. In the present case, leaving teeth 4, 12, 13, and 14 and augmenting the prosthetic posts with implants would have been an alternative. However, this would have resulted in a different prosthetic restoration. The patient’s clear desire to separate himself from his “unwanted” teeth discouraged this type of therapy. In addition, this would have resulted in a more expensive prosthetic restoration. If the premolars were retained and a telescopic prosthesis was made in the maxilla, it would have been more difficult to balance the protrusive jaw conditions.

Case reports describing stability of the implants over many years have been published [31]. On one hand, CCD can be restored with removable dentures, which are supported by implants. Alternatively, a fixed denture can be integrated with implants [32]. In this case report, the procedure and the underlying concepts of general implantology were transferred to the CCD case. The number of implants, especially in the maxilla, was chosen according to current approaches [33]. When choosing the veneering material, factors such as risk of chipping, esthetics [34], and costs must be weighed. In this case, no ceramic-veneered FDPs were incorporated for cost reasons. The screw connection of the FDPs ensures quick and cost-effective reworking. Fixed prosthetic rehabilitation was particularly important for the patient. Permanent integration of the implants is expected after the healing and loading time. The probable weak point of care here is the prosthetic care [34]. In this case, an easy to repair care was preferred. Corrections to possible fractures of veneers can be carried out much more easily here. In addition, strict adherence to the tight financial budget had to be ensured. The use of all-ceramic bridge materials in such cases still shows high failure rates. Moreover, a later change to other materials is always possible in the course of a new production. Monolithic restorations made of zirconium oxide promise a high chance of success. However, long-term data are still lacking. Long-term data on the functional load of restorations in both jaws are also not available [35]. The present case also showed good preoperative conditions for implant therapy. The high values of primary and secondary stability indicated that the bone was good for implant restoration [36]. Due to the patient’s high primary stability, it was possible to perform an immediate temporary restoration with associated immediate loading.

## Conclusion

In this case report, we described the implant-prosthetic restoration of a patient with CCD. As the 26-year-old patient had not undergone orthodontic therapy in childhood, which would have been the first-choice therapy, six implants each were placed in the maxilla and mandible and restored with acrylic resin veneered-fixed dental prostheses. This case shows that individual planning must be carried out. The clinical situation, especially the occlusion, must be considered. In addition, for patients with CCD, the emotional situation must also be incorporated into the treatment planning to a large extent. The clinical and radiological parameters observed in this case suggest that surgical and prosthetic procedure concepts from routine implantology can be adopted for patients with CCD.

## Data Availability

NA

## Abbreviations

CBCT: Cone-beam computed topography
CCD: Cleidocranial dysplasia
CBFA1: Core-binding factor subunit alpha-1
FDP: Fixed dental prostheses
RUNX2: Runt-related transcription factor 2
